# Culture-dependent decoupling of *H19* allele-specific expression from *H19/IGF2*:IG-DMR methylation in experimental models

**DOI:** 10.1080/15592294.2026.2690360

**Published:** 2026-07-30

**Authors:** Céline Selenou, Frédéric Brioude, Laure Sudre, Aurélie Pham, Irène Netchine, Marie-Laure Sobrier, Éloïse Giabicani

**Affiliations:** a Sorbonne Université, INSERM, Centre de Recherche Saint-Antoine, Paris, France; b Sorbonne Université, INSERM, Centre de Recherche Saint-Antoine, APHP, Hôpital Armand Trousseau, Endocrinologie Moléculaire et Pathologies d’Empreinte, Paris, France; c Sorbonne Université, INSERM, Centre de Recherche Saint-Antoine, APHP, Hôpital Armand Trousseau, Service de Néonatologie, Paris, France

**Keywords:** Allele-specific expression, genomic imprinting, Silver-Russell syndrome, *H19*, methylation, ICR

## Abstract

Genomic imprinting is an epigenetic mechanism leading to the monoallelic expression of a subset of genes, mainly regulated by DNA methylation at imprinting control regions (ICRs). Silver–Russell syndrome (SRS) is a paradigmatic imprinting disorder in which loss of methylation at the 11p15.5 *H19/IGF2*:IG-DMR is associated with downregulation of *IGF2* and biallelic expression of *H19*. Currently, the allele-specific expression of imprinted genes is widely inferred from ICR methylation without direct assessment. Here, we quantified *H19* allele-specific expression together with *H19/IGF2*:IG-DMR methylation in control (*n* = 32) and SRS (*n* = 12) samples spanning tissues and commonly used cellular models, including fibroblasts, induced pluripotent stem cells (iPSCs) and dental pulp stem cells (DPSCs). Overall, *H19* allele-specific expression was concordant with *H19/IGF2*:IG-DMR methylation in tissues, iPSCs and fibroblasts, with clear allelic bias in controls and predominantly biallelic expression in SRS samples. Nevertheless, some SRS-derived fibroblast lines retained an allelic bias despite loss of methylation. In addition, a subset of control DPSCs with balanced methylation exhibited biallelic *H19* expression. We showed that the observed decoupling between methylation and allele-specific expression was associated with culture-related procedures, including repeated passaging and freeze–thaw cycles in DPSCs. These findings show that ICR methylation does not always faithfully reflect imprinted gene expression and therefore cannot be uncritically used as a proxy for imprinting. They further indicate that culture-related procedures can promote loss of imprinting and support the systematic combined assessment of ICR DNA methylation and allele-specific expression when establishing cellular models for imprinting disorders.

## Background

In humans and other diploid organisms, autosomal genes are typically expressed from both parental alleles at equivalent levels. However, certain genes exhibit preferential monoallelic expression from one parental allele [1]. Genomic imprinting is an epigenetic mechanism responsible for deterministic monoallelic expression of less than 1% of genes depending on the parental origin [2,3]. Monoallelic expression of imprinted genes is mainly controlled by DNA methylation at imprinting control regions (ICRs), which are primary differentially methylated regions (DMRs). Under physiological conditions, one parental allele is methylated whereas the other remains unmethylated [4]. These ICRs co-regulate the expression of imprinted genes through associated regions called secondary DMRs, whose methylation profiles usually depend on the nearby ICR and generally vary in concert with it [5,6]. However, monoallelic expression of imprinted genes also critically depends on additional epigenetic layers, including histone modifications, higher-order three-dimensional chromatin architecture and, at some loci, imprinted long non-coding RNAs. Together, these multiple regulatory layers shape and stabilize the monoallelic expression of imprinted genes [7–9].

**Figure 1.**
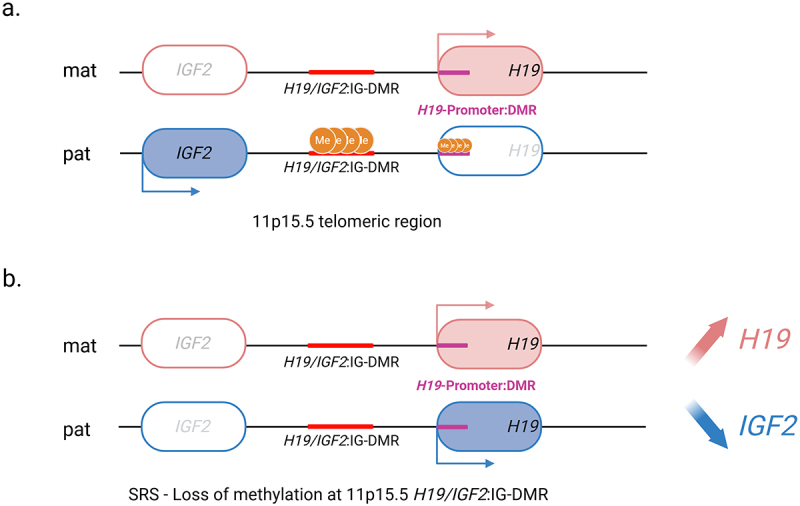
Schematic representation of the 11p15.5 chromosomal region. (a) Physiological 11p15.5 telomeric region (b) loss of methylation at *H19/IGF2*:IG-DMR in SRS. The filled box represent expressed genes, the red and purple lines represent *H19/IGF2*:IG-DMR and *H19*-Promoter: DMR, respectively. The orange circle annotated with ‘me’ represent the methylation marks. mat: maternal allele; pat: paternal allele. Created in https://BioRender.com.

Molecular defects, including loss or gain of methylation at ICRs or genetic alterations affecting imprinted loci, can arise post-zygotically and result in variable degrees of cellular mosaicism, defined by the coexistence of altered and normal cells. These defects invariably lead to abnormal expression of imprinted genes and are responsible for thirteen developmental disorders collectively known as imprinting disorders, among which Silver–Russell syndrome (SRS) represents a paradigmatic example [10]. SRS is a severe growth disorder mainly associated with loss of methylation (LOM) at *H19/IGF2*:IG-DMR (and subsequently at the secondary DMR under its dependence, *H19*-Promoter: DMR), leading to biallelic expression of *H19* and decreased expression of *IGF2*, illustrating a canonical situation in which disruption of DNA methylation directly leads to loss of imprinting at the transcriptional level (Figure 1) [11,12].

The use of experimental models to study genomic imprinting and associated disorders, particularly *in vitro* systems, has revealed a more complex relationship between DNA methylation and allelic expression. As especially documented in stem cell models, loss of imprinting can occur *in vitro* independently from detectable changes in DNA methylation [13,14]. Nevertheless, most studies working on imprinting disorder models solely rely on methylation profiling at ICRs, with no assessment of allele-specific expression of their regulated imprinted genes.

Since allele-specific expression constitutes the functional hallmark of genomic imprinting, we aimed at characterizing it in experimental samples routinely used to study the pathophysiology of imprinting disorders. To this end, we used a time-efficient and cost-effective approach to quantify *H19* allele-specific expression in a targeted manner, in parallel with *H19/IGF2*:IG-DMR methylation analysis. This method, originally developed by Lo *et al*. and previously applied in studies on dental pulp stem cells (DPSCs), is based on quantitative RT – PCR using SNP-specific probes and represents an adaptation of the TaqMan SNP genotyping technology [15,16]. By analysing a large set of control and SRS samples, we sought to identify experimental contexts that can promote decoupling between allele-specific expression and *H19/IGF2*:IG-DMR methylation.

## Materials and methods

### Biological materials

Dental pulp stem cells (DPSCs), primary skin-derived fibroblasts, induced pluripotent stem cells (iPSCs) derived from peripheral blood mononuclear cells (PBMCs), and frozen tissues (lung, muscle, kidney, and cartilage) from both controls (individuals with balanced DNA methylation at *H19/IGF2*:IG-DMR) and patients diagnosed with SRS were studied. For family trios, analyses were performed using parental DNA from leukocytes and children’s DNA and RNA from fibroblasts, DPSCs, or frozen foetal tissues. Detailed information on passage number and freeze – thaw history was available for DPSCs and iPSCs, allowing us to assess the impact of these parameters on allele-specific expression. By contrast, such information was not systematically available for primary fibroblasts, which precluded a comparable analysis in this model. A complete list of samples and associated metadata is provided in Zenodo public repository (see *Availability of data and materials*).

All cell lines used in this study had been previously generated and/or characterized in our laboratory [16–19]. PBMC collection and reprogramming into iPSCs were approved by a national *Comité de Protection des Personnes* under reference 2021-AU1597-34_IDSTEM and by the *Ministère de l’Enseignement Supérieur et de la Recherche* under authorization DC-2011–1399. Teeth collection for DPSC isolation was approved in 2016 by the Institutional Review Board (IRB 00006477) of HUPNVS, Paris 7 University, and the *Assistance Publique – Hôpitaux de Paris* (AP-HP) under reference 16–024. Some DPSCs samples have already been analysed for *H19* allele-specific expression in Giabicani *et al*. [16]. Fibroblast and tissue collection were approved by the *AP-HP* (approval no. 682) for patients recruited in France or by the Institutional Review Board (I00000204) of the Mount Sinai School of Medicine for patients recruited in the United States. Human osteoarthritic (OA) knee explants were collected as surgical waste from patients undergoing total knee joint replacement at the Maussins-Nollet Clinic (Paris, France). All experiments involving human OA samples were approved by two French Institutional Review Boards: The *Comité de Protection des Personnes Île-de-France V* and the *Comité Consultatif sur le Traitement de l’Information en Matière de Recherche*.

All experiments were conducted in accordance with French and international ethical guidelines and regulations, including the principles of the Declaration of Helsinki. Written informed consent was obtained from all patients and/or their legal guardians.

### Cell culture conditions

Skin-derived fibroblasts were cultured at 37°C with 5% CO_2_ in either: RPMI 1640 supplemented with 10% Fetal Bovine Serum (FBS) and ampicillin/streptomycin (50 U/mL and 50 µg/mL, respectively), or DMEM (1 g/L D-Glucose) supplemented with 10% FBS and 1% penicillin/streptomycin (all from Thermo Fisher Scientific, USA). Medium was renewed twice a week [17,18].

DPSCs were maintained at 37°C with 5% CO_2_ in either DMEM (1 g/L D-Glucose) or α-MEM (Thermo Fisher Scientific, USA), supplemented with 20% FBS during the first week and 10% thereafter, and 1% penicillin/streptomycin (all from Thermo Fisher Scientific, USA). Medium was renewed twice a week. For osteogenic differentiation, DPSCs were seeded in 6-well plates and cultured for 21 days in osteogenic medium consisting of DMEM (1 g/L D-Glucose) supplemented with 50 µg/mL ascorbic acid sodium salt, 10 nM dexamethasone, 10 mM β-glycerophosphate (all from Sigma-Aldrich, USA), 10% FBS and 1% penicillin/streptomycin (Thermo Fisher Scientific, USA). Medium was renewed twice a week [16].

iPSCs were generated from PBMCs of controls and SRS patients using the Sendai virus 2.0 CytoTune-iPS reprogramming kit (Thermo Fisher Scientific, USA). iPSC colonies were subsequently maintained in epiPS™ medium, consisting of mTeSR1 (STEMCELL Technologies, Canada) supplemented with 50 µg/mL ascorbic acid, on Matrigel-coated 6 cm dishes, under hypoxic conditions (5% CO_2_, 5% O_2_, 90% N_2_) at 37°C. Medium was renewed daily [19].

### DNA extraction

DNA was extracted either with a protocol after cell lysis by a salting-out as previously described for fibroblasts and leukocytes or according to the Kit Nucleospin Tissue XS for other cell types (Macherey-Nagel™, Germany) [20].

### Genomic DNA sequencing

The presence of a heterozygous polymorphism in the *H19* gene was confirmed in genomic DNA samples. A region containing two common polymorphisms – rs217727 and rs10840159 (minor allele frequencies of 0.19 and 0.45, respectively) – was amplified using the following primers: forward 5′-CAGTCACCCGGCCCAGAT-3′ and reverse 5′-AAGACACCATCGGAACAGCA-3′. The amplified products were then sequenced by standard Sanger sequencing (Eurofins Genomics, Germany), and electrophoregrams were analysed using Chromas 2.6.6 software (Technelysium Pty Ltd, Australia).

### DNA bisulfite treatment

Sodium bisulfite treatment generates an artificial C/T polymorphism by converting unmethylated cytosine to uracil residues. This process is used to distinguish the methylated and unmethylated allele. Genomic DNA (300ng) was treated with sodium bisulfite using the EZ DNA Methylation Lighting kit (Zymo Research, USA), according to the manufacturer’s instructions. Bisulfited genomic DNA was eluted in 30 µL of RNAse-Free water and stored at −20°C.

### TaqMan allele-specific methylated multiplex real-time quantitative PCR (ASMM RTQ-PCR) and methylation analysis

The methylation levels of *H19/IGF2*:IG-DMR and *H19*-Promoter: DMR were assessed by ASMM-RTqPCR, as previously described by Azzi *et al*. [21]. The methylation index (MI) at each locus was assigned by calculating the ratio between the methylated and unmethylated alleles as follows: (amount of methylated allele/sum of both methylated and unmethylated alleles) × 100. The probes and primers used are listed in Table SD1.

### RNA extraction and reverse transcription

Total RNA was extracted from the different cell-types using the NucleoSpin miRNA Kit for the isolation of small and large RNA (Macherey – Nagel, France) with DNase treatment. Both DNA and RNA were quantified using a DS-11 spectrophotometer (DeNovix, USA). cDNA was synthesized from long RNAs (600 ng) using Superscript II (Thermo Fisher Scientific, USA) and used for Allele Specific Expression (ASE)-RT-qPCR.

### ASE-RT-qPCR

ASE-RT-qPCR was performed using the QuantStudio™ 3 Real-Time PCR System (Thermo Fisher Scientific, USA) in a 96-well plate. Each well had a final reaction volume of 20 µL, containing 24 ng of cDNA (4 µL per well), TaqPath™ ProAmp™ Master Mix (Thermo Fisher Scientific, USA), and a TaqMan® SNP Genotyping Assay targeting *H19* rs217727 (Assay ID : C__2603707_10; sequence context : AACCGTCC[A-VIC/G-FAM]CCGCA, Thermo Fisher Scientific, USA) and *H19* rs10840159 (Assay ID : C___2603706_10; sequence context : GGTGGCC[A-VIC/G-FAM]TGAAG, Thermo Fisher Scientific, USA). All samples were run in triplicate. Amplification conditions and reaction mixture compositions for each SNP are detailed in Table SD2. The standard curves were established using the genomic DNA of a heterozygous individual for each targeted SNP. Therefore, it is assumed that each probe will bind equivalently to each allele. This genomic DNA was initially used at a concentration of up to 25 ng/µL, followed by one in five serial dilutions repeated four times to establish a standardized range (25 ng/µL, 5 ng/µL, 1 ng/µL, 0.2 ng/µL, 0.04 ng/µL, corresponding to 100 ng, 20 ng, 4 ng, 0.8 ng, and 0.16 ng per well, respectively). The correlation coefficient (R^2^) of standard curves which associate fluorescence to the amount of the allele for each probe is greater than 0.98. The allele-specific expression for each SNP (rs217727 or rs10840159) was determined by calculating the ratio between the [A] allele and the [G] allele, presented as a percentage of [A]: (amount of [A] allele / sum of both [A] and [G] alleles) × 100.

### Statistical analysis

All graphs and statistical analyses were performed using Prism 10.6.1®. Data are presented as means with standard deviations (SD). Non-parametric tests were used for analyses except for the linear regression tests.

## Results

### Study samples and experimental materials

A total of 56 samples were included in this study, comprising 41 controls and 15 SRS samples, spanning foetal to adult tissues, primary fibroblasts, iPSCs and DPSCs. Among these, 32 control and 12 SRS samples were heterozygous for at least one informative *H19* SNP and could therefore be analysed for allele-specific expression. Primary fibroblasts and DPSCs were analysed between passages 0 and 7 and cultured under standard conditions, with DPSCs also examined after osteogenic differentiation. iPSC lines were analysed between passages 2 and 13 and maintained in epiPS™ medium under hypoxic conditions (See *Materials and methods: Cell Culture Conditions*). In addition, frozen adult and foetal tissue samples (lung, kidney, muscle and cartilage) were included. An overview of the experimental workflow is provided in Figure SD1. The distribution of control and SRS samples across tissues and cellular models is detailed in Table SD3.

### Technical validation

**Figure 2.**
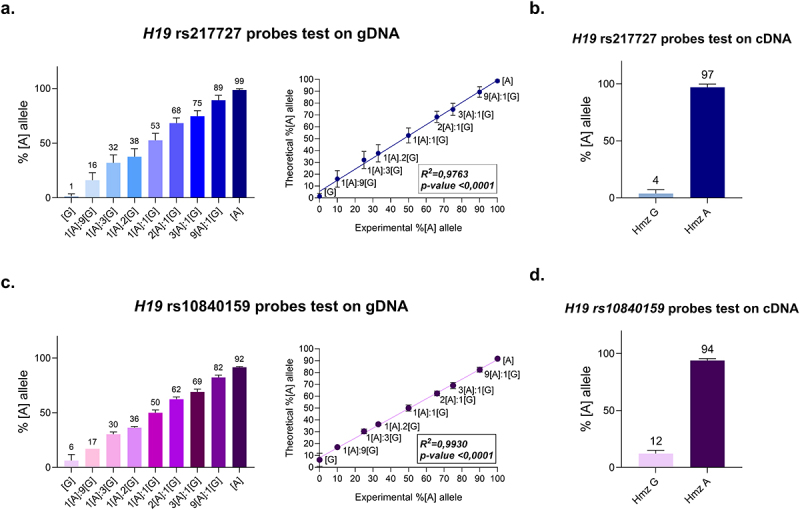
Analytical performances of probes for *H19* SNPs rs217727 and rs10840159. (a,c) Experimental quantification of the [A] allele percentage using ASE-RT – qPCR on genomic DNA (gDNA) across defined mixtures of homozygous [A] and [G] DNA for SNPs rs217727 (a) and rs10840159 (c). Data are shown as mean ± SD from three independent experiments (*n* = 3). Linear regression analysis was performed to assess the quantitative performance of each probe set. (b,d) evaluation of allele-specific detection using the same probe sets on cDNA from individuals homozygous for either the [A] or [G] allele at rs217727 (b) and rs10840159 (d). Data are shown as mean ± SD. Hmz: homozygote.

Firstly, we evaluated the diagnostic performance of probes targeting the *H19* SNPs rs217727 and rs10840159, both of which exhibit an A/G polymorphism. To this end, we compared the experimentally measured percentage for A allele with the theoretical values expected from defined mixtures of homozygous [A] and [G] genomic DNA. Linear regression analysis demonstrated excellent linearity for both probe sets, with R^2^ values of 0.976 for rs217727 and 0.993 for rs10840159, confirming accurate quantification across the full allelic range (Figure 2(a,c), SD2). Probe specificity was further assessed using complementary DNA from homozygous individuals. Each probe set predominantly detected the corresponding allele, with minimal non-specific signal (Figure 2(b,d)).

### *Parent-specific monoallelic expression of imprinted gene* H19

**Figure 3.**
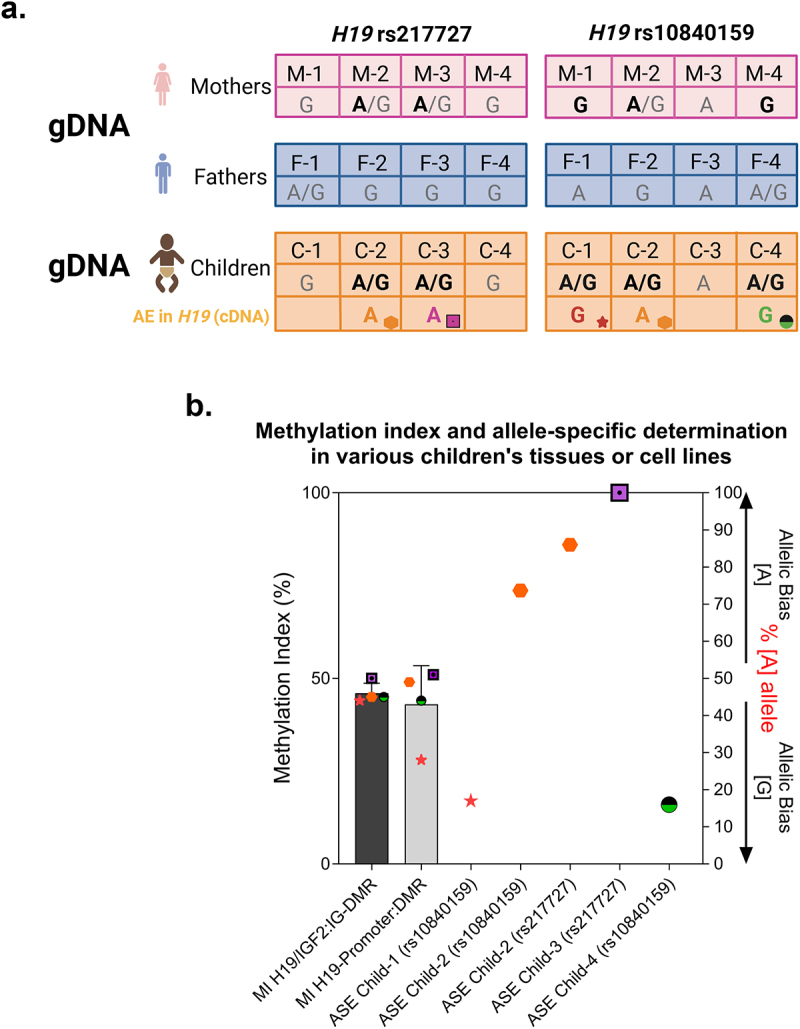
Maternal allele-specific expression of H19 in family trios. (a) Summary of parental and offspring genotypes determined by Sanger sequencing of *H19* exon 1, together with the allele-specific expression (ASE) of *H19* from the maternal allele. Created in https://BioRender.com. (b) Assessment of DNA methylation and allele-specific expression in fibroblasts from child-1 and child-2, kidney from child-3, and dental pulp stem cells at passage 2 from child-4. Black and grey bars represent the methylation index at the *H19/IGF2*: IG-DMR and the *H19*-Promoter: DMR, respectively, in the four individuals. Methylation data are shown as mean ± SD. Symbols refer to panel (a) and indicate *H19* allele-specific expression at SNPs rs217727 and rs10840159 measured by ASE-RT – qPCR in cDNA from the four children. MI: methylation index.

We tested whether *H19* is expressed from the maternal allele, as physiologically expected. We analysed four family trios informative for one or both SNPs: each child had parents with distinguishable alleles and showed normal methylation at *H19/IGF2*:IG-DMR, classifying them as controls (Figure 3). In all cases, the predominantly expressed allele corresponded to the one inherited from the mother, confirming maternal-specific monoallelic expression of *H19* (Figure 3(b)).

### H19 *allele-specific expression in tissues and cell-based models*

**Figure 4.**
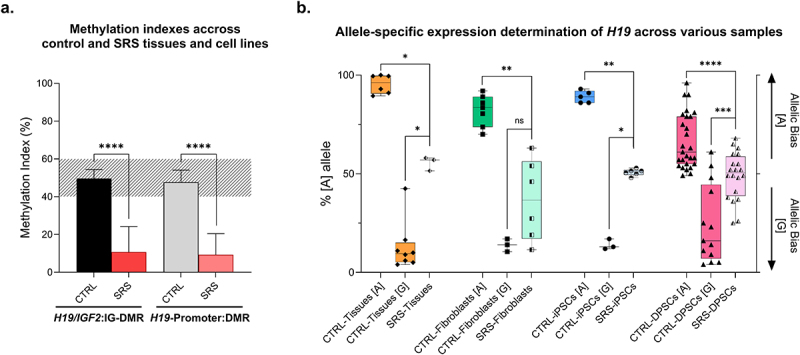
Methylation levels at *H19/IGF2* locus and allele-specific expression of *H19* across various samples from both control individuals and patients with SRS. (a) Methylation indexes (MI) in controls and SRS tissues and cell lines at *H19/IGF2*: IG-DMR and *H19* -Promoter: DMR. The hatched part represents the average normal MI area for each locus. Data are shown as mean ± SD. (b) The allele-specific expression of *H19* was assessed in various tissues (cartilage, lung, kidney and muscle) and cultured cell types (fibroblasts, DPSCs and their osteogenic differentiation and iPscs). Results combine data from the two *H19* SNPs, rs10840159 and rs217727. The results by SNPs are available in *Figure SD4*. For cultured cells, control samples were classified based on the allelic bias observed at the earliest available passage: samples with a low %A value were categorized as [G]-biased, and those with a high %A value as [A]-biased. This classification was maintained even if allelic expression drifted toward biallelic expression at later passages. Each sample type is represented by a specific symbol and colour. Control samples are shown with darker colours and solid symbols, while SRS samples are represented by lighter colours and half-filled symbols. Data are shown as box-and-whisker plots (min to max), with the box representing the interquartile range and the line indicating the median. All statistical analyses were performed using the mann – Whitney U test. *p*-values: *p* < 0.05 (*), p < 0.01 (**), *p* < 0.001 (***), *p* < 0.0001 (****); ns: non-significant.

We broadened our analysis of *H19* allele-specific expression by testing control (*n* = 32) and SRS (*n* = 12) samples derived from various sources, including foetal and adult frozen tissues, cultured fibroblasts, iPSCs and DPSCs. All samples used for allele-specific expression analysis were heterozygous for either *H19* rs10840159, *H19* rs217727, or both and displayed expected methylation profiles (Figure 4(a), SD3).

In frozen tissues, control individuals generally exhibited a strong allelic bias consistent with monoallelic maternal expression of *H19*, although one control sample showed predominantly biallelic expression. In contrast, SRS tissue samples consistently displayed biallelic expression (Figure 4(b)).

In fibroblasts, control samples exhibited a clear allelic bias, consistent with monoallelic expression of *H19*. Among SRS fibroblast samples, some displayed biallelic expression, while others retained an allelic bias. As a result, a significant difference in allelic expression patterns was observed between SRS and [A]-biased control group, but not the [G]-biased one (Figure 4(b)).

In iPSCs, control lines exhibited a clear allelic bias toward either the [A] or [G] allele. In contrast, SRS iPSC lines displayed a biallelic expression of *H19*, consistent with a loss of imprinting secondary to LOM at the *H19/IGF2:IG-DMR* (Figure 4(b)).

Conversely, DPSCs were the cell-type displaying the most variable allele-specific expression patterns in control samples, with some displaying weak or no allelic bias. In parallel, SRS samples tended to exhibit predominantly biallelic expression (Figure 4(b)).

Finally, to investigate whether the degree of cellular mosaicism for loss of methylation at *H19/IGF2*:IG-DMR was associated with the level of *H19* allele-specific expression in SRS samples, we performed a linear regression analysis across iPSCs, fibroblasts, DPSCs and foetal tissues. No significant correlation was observed between methylation indexes and allele-specific expression (R^2^ = 0.0004, Figure SD5).

### Impact of cell culture – associated procedures on H19 allele-specific expression in DPSCs

**Figure 5.**
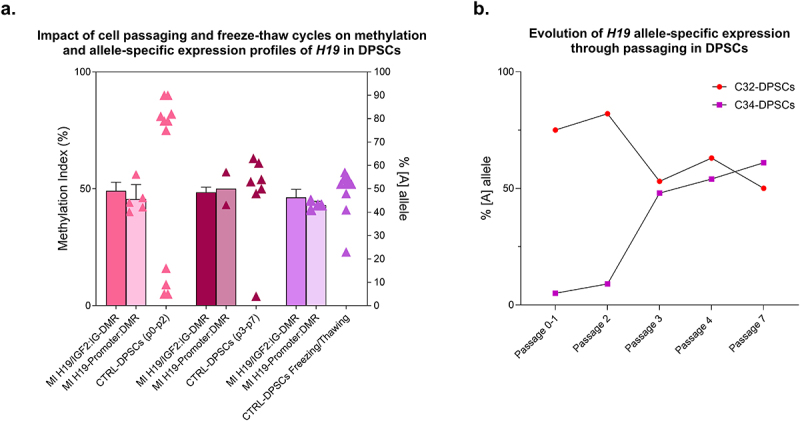
Impact of cell passaging and freeze-thaw cycles on methylation and allele-specific expression of *H19* in control dental pulp stem cells (DPSCs). (a) Methylation index (MI) at the *H19/IGF2* :IG-DMR and, where available, at the *H19* -Promoter: DMR together with *H19* allele-specific expression (rs10840159 and rs217727) assessed by ASE-RT-qPCR were analysed in control DPSCs under different conditions. Samples classified as ‘p0-p2’ (early passages) or ‘p3-p7’ (late passages) referred exclusively to cells that were never frozen. Samples classified as ‘freezing/thawing’ corresponded to DPSCs that underwent cryopreservation and thawing, regardless of their passage number. Methylation data are shown as mean ± SD. (b) *H19* allele-specific expression assessed by ASE-RT-qPCR across successive passages in two control DPSC lines (C32 and C34).

Given the heterogeneity of *H19* allele-specific expression observed among control DPSCs, we sought to investigate its underlying causes. Further analysis revealed that samples examined beyond passage 2 or following freeze – thaw cycles often exhibited a progressive loss of allelic bias. Notably, this shift occurred despite the maintenance of normal methylation profiles at both *H19/IGF2*:IG-DMR and *H19*-Promoter: DMR (Figure 5(a), SD3d). To illustrate this, we selected two control DPSC samples and followed them across successive passages, demonstrating a progressive erosion of allelic bias beyond passage 2 (Figure 5(b)).

## Discussion

In this study, we assessed *H19* allele-specific expression with *H19/IGF2*:IG-DMR methylation across a broad panel of human tissues and cellular models commonly used to study imprinting disorders. We show that, although *H19* allele-specific expression is largely concordant with *H19/IGF2*:IG-DMR methylation in most of experimental materials, it can be disrupted in specific contexts. In particular, we identified decoupling between methylation profiles and allele-specific expression in control DPSCs, indicating that DNA methylation alone is not sufficient to infer allelic expression of imprinted genes in this experimental model.

In this study, we deliberately focused our allele-specific expression analyses on *H19* rather than on *IGF2*, although both genes are co-regulated by the same imprinting control region. This choice was motivated by both biological and technical considerations: *IGF2* is frequently downregulated in SRS samples, which limits the sensitivity and robustness of allele-specific expression measurements, and it harbours very few common SNPs, resulting in a low proportion of informative samples [22]. In contrast, *H19* is either overexpressed or stably expressed in SRS and contains several frequent SNPs with high minor allele frequencies in the general population, according to public databases [23,24]. This enables robust quantitative assessment of allelic expression in a large fraction of samples. Beyond these considerations, the use of ASE RT – qPCR to assess allele-specific expression offers practical advantages, as it relies on a simple and widely accessible RT – qPCR – based workflow. Moreover, numerous commercial SNP genotyping assays are available for a wide range of common polymorphisms, including at many imprinted loci, making this strategy theoretically applicable to other genes and experimental materials. Although most results on samples of interest were consistent with physiological expectations, we identified some unforeseen findings. The discordance between methylation profiles and allele-specific expression patterns in some control and SRS samples raises important concerns regarding the stability of imprinting across different experimental cells and tissues. Understanding the biological origins of such allele-specific expression variability would be useful to improve the relevance of cellular models in the field of research on imprinting disorders.

One possible explanation for this discordance could be the low *H19* expression in some samples. However, Baran *et al*. have shown that *H19* maintains monoallelic expression in a wide array of tissues, including skin, suggesting that allelic bias should remain detectable even at low expression levels [25]. Supporting this, in our own dataset, some control iPSC and fibroblast samples with relatively low *H19* expression still exhibited clear allelic bias, indicating that detection of allelic imbalance remains reliable under such conditions. Alternatively, procedures linked to *in vitro* culture could underlie this discrepancy. Cell culture conditions are known to impact methylation profiles and transcriptional activity including genomic imprinting [13,26–28]. In this regard, our study showed an almost systematic loss of imprinting in control DPSCs secondary to passages or freeze – thaw cycles, without any associated methylation abnormalities – highlighting a discordance between methylation and allele-specific expression. Interestingly, cell culture conditions did not significantly compromise *H19* allelic expression in control fibroblasts or iPSCs. These findings suggest that *in vitro* manipulation of DPSCs can compromise imprinting stability, leading to a drift toward biallelic expression of *H19* in some cell types. In addition to culture-related effects, mosaicism may also contribute to unexpected allelic patterns. In this context, a methylation index (MI) close to 0% reflects a large cell population with LOM, whereas MI closer to 50% indicate increasing mosaicism between unmethylated and normally methylated cells. This may explain why some SRS fibroblast samples exhibited a clear allelic bias, rather than the expected trend toward biallelic expression. However, this interpretation is not fully supported by our data, as we observed low MI in fibroblasts from SRS patients that nonetheless exhibited a clear allelic bias. This indicates a high proportion of cells with epigenetic defects within the tissue, yet without the expected shift toward biallelic expression. These observations suggest that there is no direct correlation between MI and *H19* allele-specific expression in these cases. Nevertheless, due to the absence of parental genotypes, we cannot definitively assess the contribution of cellular mosaicism to the observed allelic expression patterns in SRS samples. Alternatively, culture adaptation mechanisms could underlie these discrepancies. Since *H19* acts as a growth suppressor, its biallelic expression may be disadvantageous for cell proliferation *in vitro* [29]. Selection pressures in culture might therefore favour cells with reduced or monoallelic *H19* expression, independent of methylation status at the ICR or secondary DMRs. This suggests that transcriptional regulation of *H19* can, under certain conditions, become uncoupled from DNA methylation in some samples.

These examples highlight that, although it may appear that we fail in clearly discriminating between control and SRS samples in a few specific cases, this limitation is unlikely due to technical shortcomings. On the contrary, the high analytical performance demonstrated by the method supports its robustness and reliability. Therefore, the observed discrepancies more likely reflect true biological complexity – specifically, alterations in the regulation of *H19* allelic expression in certain samples. These discordances between allelic expression and DMR methylation profiles may reveal the influence of additional regulatory layers – such as histone modifications at imprinted loci – that could be modulated by *in vitro* culture conditions, freeze – thaw stress, or other environmental clues [30].

In parallel, allele-specific expression proved particularly robust in iPSCs, a notable achievement considering that iPSCs cultured under standard conditions often exhibit epigenetic abnormalities, especially within imprinted regions [19, 31–33]. These defects are typically observed at paternally methylated DMRs like the *H19/IGF2*:IG-DMR, where aberrant gains in methylation are associated with biallelic expression of imprinted genes they control [19, 31–33]. However, under improved culture conditions, expected methylation profiles can be preserved in both control and SRS-derived iPSCs at these DMRs [19]. In this study, we report for the first time the biallelic expression of *H19* in SRS patient-derived iPSCs, providing a functional readout that directly reflects the underlying epigenetic defect. By contrast, control iPSCs with preserved methylation at the *H19/IGF2*:IG-DMR exhibit clear monoallelic expression of *H19*, confirming imprinting stability. Unlike DPSCs and fibroblasts, iPSCs undergo clonal expansion, which minimizes cellular heterogeneity and may contribute to the systematic concordance observed between methylation status and allele-specific expression. These data confirm that these new culture conditions and differentiation protocols do not affect the genomic imprinting process in these cells, making them relevant to model imprinting disorders.

## Conclusion

In conclusion, although allele-specific expression is generally consistent with DNA methylation profiles in samples, this study highlights that methylation status alone may not fully reflect genomic imprinting. Indeed, we show that loss of imprinting can emerge in specific biological and experimental contexts – including culture conditions – without detectable methylation abnormalities. By integrating allele-specific expression analysis alongside DNA methylation profiling, we propose a more comprehensive framework to assess genomic imprinting integrity. This dual approach refines the selection of cell lines with preserved genomic imprinting for *in vitro* studies and provides a complementary tool for assessing the functional impact of methylation alterations. These findings add a novel layer of complexity to the interpretation of parental imprinting regulation, particularly in *in vitro* models, and should be carefully monitored in future studies on genomic imprinting.

## Open scholarship

This article has earned the Center for Open Science badge for Open Data. The data are openly accessible at https://doi.org/10.5281/zenodo.17388208

## Availability of data and materials

All data supporting the findings of this study are available from the corresponding author upon reasonable request. Processed datasets have also been deposited in the Zenodo public repository (https://doi.org/10.5281/zenodo.17388208).
